# Progress of Surgery

**Published:** 1900-12-08

**Authors:** 


### Progress of Surgery.

### HERNIA.

**Anatomy of the Inguinal Region.**—Joseph A. Blake[1] maintains that many writers misapply the term "conjoined tendon" in describing operations for the radical cure of inguinal hernia. He states that a careful dissection shows that as a rule the lowermost fibres of the internal oblique do not enter into the formation of the conjoined tendon, but pass from their origin in Poupart's ligament to be inserted in front of the rectus. The conjoined tendon is chiefly derived from the deeper aspect of the internal oblique, and slightly from the transversalis muscle. It consists of a few muscular fasciculi arching over the cord to be inserted into the ileo-pectineal line. It lies in front of the main aponeuroses of the transversalis muscle, the fibres of the latter passing behind the conjoined tendon to unite with the internal oblique in front of the rectus. Blake has carefully examined the conjoined tendon on both sides in twenty-five normal muscular subjects. He found that in none of these cases did the tendon extend more than five-eighths of an inch laterally from the insertion of the rectus, and in the majority the extension was less than half an inch. There was, however, always a distinct posterior wall to the inguinal canal formed by the thickened transversalis fascia. The aponeurosis of the transversalis muscle is deficient for a short distance above Poupart's ligament in the region of the inguinal canal, and the structure referred to as the transversalis fascia is the only protection in this part of the abdominal wall after the removal of the internal and external oblique muscles. It is this fascia, called an aponeurosis by Douglass, that Blake considers many writers have mistaken for the conjoined tendon. The description in *Quain's Anatomy* is alleged to be based on Douglass' article, and refers to this fascia or aponeurosis as a part of the conjoined tendon. Blake then points out that neither this fascia nor the true conjoined tendon are in reality utilised in operations for radical cure, the latter not being used because it is such an insignificant structure. In Bassini's operation the main reliance has to be placed upon the internal oblique muscle, and the effort should be made to restore the normal parallelism of its fibres to Poupart's ligament. It is futile to attempt to draw down the transversalis, as it is normally deficient for a considerable distance above the ligament. It is not recommended as a routine procedure to suture the border of the rectus to Poupart's ligament. Bassini recommended it in some cases. Wöfler's method of doing this by opening the anterior layer of the sheath of the rectus is deprecated, as it severs the insertion of the internal oblique and transversalis, and so weakens the abdominal wall. Bloodgood opens the sheath posteriorly.

**The Radical Cure of Inguinal Hernia.**—Frederick D. Bird[2] has written on this subject. Although a strong adherent of Bassini's procedure, Bird considers it unsurgical to follow all its details in every case. He considers a good inch of the skin incision, usually made towards the pubes, is unnecessary, and that often it is not necessary to incise the skin as far as the external ring, in which case one or two fair-sized arteries and veins escape division. He extends his incision well beyond the site of the internal ring, and makes it well above Poupart's ligament, entering a region which allows of marked mobility. The ligament is analogous to a bony margin, and therefore the further the scar is kept away from it the better. A full inch distance is recommended. The deep layer of the superficial fascia should not be stripped widely from the aponeurosis beneath, as it has vascular connections with it. In old herniæ this fascia attains strength, and may be utilised as an accessory restraining agent. The division of the external oblique aponeurosis should be at least half an inch above Poupart's ligament in order to leave a good fringe attached to that structure, facilitating the deep suturing. A linear hiatus in the aponeurosis often shows where it is best to make the split. It is wrong to cut fibrous structures if it can be avoided. The external ring should not be divided if it is possible to carry out the necessary manipulations without its division. More room may be got by carrying the incision in the aponeurosis an inch or two beyond the internal ring. In other cases the incision may be made above the external ring and the latter turned down with the lower flap. The former plan is much better. The eligibility of any case for radical cure is considered to depend upon the perfection of the internal oblique muscle in the region concerned more than upon anything else. In an ideal case the muscle forms a complete curtain, and in other good cases there is a well-defined lower edge to the muscle, but in the worst cases there are very few muscular fibres arising from Poupart's ligament, no lower arciform edge can be found, and the muscle present is cremasteric. Any cutting of the internal oblique is most mischievous. The cord is then lifted up by a finger, passed behind it, and spread out so that the denser tissue of the sac can be seen and attacked from above and behind. The cord need not be entirely dislocated from its bed. By this means the nerves of the cord are preserved. The sac is much more easily eliminated from the cord by beginning high up near the deep epigastric artery. After ligature of the sac, if a satisfactory muscular curtain exists, Bird considers it harmful to pass the deep Bassini sutures. Without them the operation secures almost perfect results. A. M. Phelps, of New York,[3] has described his operation for the radical cure of hernia. He predicts that the day is not far distant when Bassini's operation, and all others, except the one he describes, will be abandoned by every operator. The special features of the operation are the introduction of a mattress of silver wire, to fortify the inguinal canal, and stitching the muscular layers over this, entirely obliterating the canal, and bringing the cord out immediately underneath the skin. The sac is not ligatured—it is cut off—and the opening left is sutured as in any other abdominal operation. The suture used for the peritoneum and the transversalis fascia is a continuous one of silver wire, and the muscles are similarly sutured over the wire mattress. A small glass drainage tube is inserted down to the mattress. The cord is brought out externally and inferiorly to the internal abdominal ring, the apeneurosis of the muscles being notched to prevent strangulation. Phelps says he knows of no other operation which safeguards the operator against the possibilities of relapse; that there is no danger from irritation of the wire; and his mortality has been nil. The mattress and the fibrous tissue produced around it will bend with every movement of the body. The use of silk is positively contra-indicated on account of the dangers of infection. The wire is taken from pure carbolic and heated to redness in an alcohol lamp. If suppuration should occur the wire should by no means be removed, but the wound laid open, all the infected portion curetted out, the wound filled with pure carbolic acid, and afterwards washed out with alcohol. The results in the infected cases are equally as good as in the non-infected. The principle of this operation may be applied in repairing any divided muscle, and for large cranial wounds. Since 1892 Phelps has operated upon 216 cases of hernia: 46 were relapsed Bassinis, and 51 relapses from other operations. Suppuration followed in 16 cases and was treated as described above. There were no relapses. The use of buried wire sutures is advocated also by Albert H. Tuttle[4] of Cambridge, Mass. This surgeon uses chromicised tendon in ventral hernia and in children, and silver wire in umbilical, femoral, and inguinal hernia of the adult. Large-sized wire is used with a needle attached at each end, and it is applied "after the manner of cross-lacing a shoe, except that the wire crosses itself, first on the inside, and then on the outside. Each time the wire crosses outside, that is in the superficial part of the wound, two or three twists are taken, which secures the sutures at this point and facilitates the work of lacing."

[1] Med. Rec., Vol. 58, No. 9. [2] Lancet, Aug., 1900. [3] Med. Rec., Vol. 58, No. 12. [4] Med, Times and Register, June, 1900.

